# Fine-Tuning the Antimicrobial Profile of Biocompatible Gold Nanoparticles by Sequential Surface Functionalization Using Polyoxometalates and Lysine

**DOI:** 10.1371/journal.pone.0079676

**Published:** 2013-10-17

**Authors:** Hemant K. Daima, P. R. Selvakannan, Ravi Shukla, Suresh K. Bhargava, Vipul Bansal

**Affiliations:** NanoBiotechnology Research Laboratory, School of Applied Sciences, Royal Melbourne Institute of Technology University, Melbourne, Victoria, Australia; Dowling College, United States of America

## Abstract

Antimicrobial action of nanomaterials is typically assigned to the nanomaterial composition, size and/or shape, whereas influence of complex corona stabilizing the nanoparticle surface is often neglected. We demonstrate sequential surface functionalization of tyrosine-reduced gold nanoparticles (AuNPs^Tyr^) with polyoxometalates (POMs) and lysine to explore controlled chemical functionality-driven antimicrobial activity. Our investigations reveal that highly biocompatible gold nanoparticles can be tuned to be a strong antibacterial agent by fine-tuning their surface properties in a controllable manner. The observation from the antimicrobial studies on a gram negative bacterium *Escherichia coli* were further validated by investigating the anticancer properties of these step-wise surface-controlled materials against A549 human lung carcinoma cells, which showed a similar toxicity pattern. These studies highlight that the nanomaterial toxicity and biological applicability are strongly governed by their surface corona.

## Introduction

Most of the pathogenic bacterial strains have developed resistance toward available pharmaceutical compounds through genetic mutations and it is an issue of increasing concern for public health [1,2]. Recently, it has been realized that inorganic nanomaterials have potential to be used as antimicrobial agents as they demonstrate composition, size, shape, chemical functionality and surface charge-dependent antimicrobial profile towards controlling pathogenic microorganisms. In this context, predominantly silver nanoparticles (AgNPs) are found to exhibit significantly higher level of toxicity [3-6], whereas gold nanoparticles (AuNPs) are considered highly biocompatible as demonstrated by us and others previously [3,7,8]. The antibacterial activity of AgNPs is observed to be size dependent [9,10], wherein metal nanoparticles of 1-10 nm diameter are considered to exhibit a direct interaction with biological systems and do not show much variation in their biological profile within this size range [11]. Moreover, AgNPs are known to undergo a shape-dependent interaction with the Gram negative bacterium *E. coli*, and therefore AgNPs shape also contributes to their antimicrobial profile [12]. 

In contrast to composition, size and shape-dependent antimicrobial studies, the influence of nanoparticles surface corona on their antibacterial action has not been well-studied systematically, and is rather unclear. It is typically believed that peripheral coatings onto nanoparticles surface may assist in controlling the surface charge of nanoparticles and therefore allow appropriate electrostatic interactions between nanoparticle and the biological surface [13-15]. Importance of amphiphilic amino acids towards adsorbing proteins on nanopatterned surfaces was also recently established through molecular dynamic simulation studies [16]. In an interesting study, Rotello et al demonstrated that cationic AuNPs may exhibit moderate toxicity by causing cell lysis, whereas anionic AuNPs were found to be nontoxic [17]. This undoubtedly indicates that even the most biocompatible nanomaterials such as AuNPs can be made toxic by tuning its surface properties such as surface charge and chemical functionality. The role of surface charge switching in polymer nanoparticles toward cell wall-targeted delivery of antibiotics was also discussed [18].

In the current study, we discuss our efforts on step-wise control of the antimicrobial profile of tyrosine-reduced AuNPs^Tyr^ by employing a sequential surface functionalization strategy using anionic polyoxometalates (POMs) and cationic lysine molecules. AuNPs were chosen as a model system of choice because of their known biocompatibility and suitability for a range of biological applications. POMs were chosen for surface functionalization because POMs are widely recognized in the field of medicine due to their antibacterial, antiviral and anticancerous activities [19-21]. Notably, POMs are highly negatively charged clusters of Keggin ions consisting of early transition metals and oxygen atoms formed by self-assembly processes [22,23], however most of the POMs are unstable in aqueous solutions at physiological pH, which limits their medicinal and biological applications [19]. The stability of POMs could be increased by anchoring them on to biocompatible AuNPs^Tyr^ surface, as we recently demonstrated in the case of silver nanoparticles [15]. More specifically, we synthesized AuNPs^Tyr^ using a novel green approach involving tyrosine amino acid, followed by their surface functionalization using two different POMs viz. 12-phosphotungstic acid (PTA) or 12-phosphomolybdic acid (PMA), which was further followed by modification of these nanomaterials using cationic amino acid lysine to step up their toxicity in a controllable manner. Antibacterial activities of these functionalized nanomaterials were evaluated against a model bacterium *E. coli*. By varying the level of surface functionalization of biocompatible AuNPs^Tyr^ with POMs and lysine, we were able to achieve different levels of antibacterial activity in a highly controlled fashion. Major advantage of this approach is that AuNPs^Tyr^ act as a carrier and stabilizer for the antimicrobial component (POMs), whereas the presence of the cationic amino acid lysine in the outermost shell assists in directing these nanomaterials toward negatively charged bacterial cells. The generality of our observations was further affirmed by investigating the influence of these sequentially surface-functionalized coatings on mammalian lung carcinoma cells. The current study therefore strengthens the importance of nanomaterial surface functionality as the driving force to fine-tune the antimicrobial and other biological properties of nanomaterials.

## Materials and Methods

### Reagents and Materials

Tetrachloroauric acid (HAuCl_4_), L-tyrosine (Tyr), phosphotungstic acid (PTA) and potassium hydroxide (KOH) were purchased from Sigma-Aldrich and phosphomolybdic acid (PMA) was purchased from Chem-Supply Pty Ltd. Dialysis tubing cellulose membrane (12 KDa molecular weight cut-off) was purchased from Sigma-Aldrich and used after processing (boiling twice for 15 min in deionized MilliQ water). The model bacterial organism (*E. coli* DH 5α) used in this study was bought from Southern Biological and the A549 human lung carcinoma cells were acquired from ATCC. Molecular Biology grade nutrient agar (NA) and Luria-Bertani broth (LB) were purchased from Oxoid and US Biologicals, respectively, and used to grow and maintain the bacterial culture as per the standard protocol. All the solutions were prepared using deionized (MilliQ) water.

### Tyrosine-mediated synthesis of AuNPs^Tyr^

In a typical experiment, 300 mL aqueous solution consisting of 0.1 mM L-tyrosine and 1 mM KOH were allowed to boil. Under alkaline boiling conditions, HAuCl_4_ was added to the above solution, resulting in 0.2 mM equivalent of gold ion concentration. The above solution was further boiled for 5 min, which resulted in a ruby-red color solution consisting of tyrosine-reduced AuNPs^Tyr^. To increase the metal concentration by a factor of three, this AuNPs^Tyr^ solution was further boiled to reduce the volume to 100 mL. This colloidal solution was found highly stable even after concentration, signifying that AuNPs^Tyr^ were strongly capped by tyrosine amino acid. Further, concentrated AuNPs^Tyr^ solution was dialyzed three times against deionized water using cellulose dialysis membrane to remove the excess KOH, potentially unreduced metal ions and unbound amino acid, if any. 

### Sequential surface functionalization of AuNPs^Tyr^ with POMs and L-lysine

Concentrated and dialyzed AuNPs^Tyr^ were surface functionalized with two different POMs viz. PTA and PMA, followed by L-lysine amino acid. For functionalization of AuNPs^Tyr^ with POMs, concentrated AuNPs^Tyr^ were separately mixed with PTA or PMA aqueous solutions to achieve 0.1 mM POM concentration and incubated for 24 h. Following incubation, these solutions were again subjected to dialysis to remove uncoordinated PTA or PMA molecules, thereby resulting in POM functionalized AuNPs (AuNPs^Tyr@PTA^ and AuNPs^Tyr@PMA^). Consequently, AuNPs^Tyr@PTA^ and AuNPs^Tyr@PMA^ were further modified with a cationic amino acid lysine by adding L-lysine to these solutions to the final lysine concentration 0.1 mM, incubating for 24 h, followed by dialysis to remove uncoordinated lysine molecules, which resulted in lysine-functionalized nanomaterials viz. AuNPs^Tyr@PTA-Lys^ and AuNPs^Tyr@PMA-Lys^. 

### Antibacterial assays of POMs and lysine-functionalized AuNPs

Quantitative assessment of antibacterial potential of AuNPs^Tyr^, AuNPs^Tyr@PTA^, AuNPs^Tyr@PMA^, AuNPs^Tyr@PTA-Lys^ and AuNPs^Tyr@PMA-Lys^ nanomaterials was performed using colony counting method. In the colony counting method, 1 x 10^4^ cells of Gram negative bacterium *E. coli* were incubated with various concentrations (dosages) of extensively dialyzed NPs in 1 mL LB medium for 15 min. Dialysis was considered essential to ensure that the observed antibacterial action is due to nanomaterials, wherein potentially unreduced metal ions, free amino acids and POMs do not contribute to the antimicrobial profile. Following incubation, 100 μL aliquots were plated on to NA plates and bacterial colonies grown overnight at 37 °C were counted, which corresponded to the number of live bacteria (colony forming units – CFUs) in each suspension as a result of interaction of different NPs with bacteria for 15 min. The viability of *E. coli* versus dosage of different AuNPs (concentrations of tungsten (W) or molybdenum (Mo) present in functionalized AuNPs^Tyr@POMs^ or AuNPs^Tyr@POMs-Lys^) was plotted to assess the effect of surface functionalization on antimicrobial profile. It may be noted that in AuNPs^Tyr^ sample, which did not have either W or Mo, the highest equivalent amount of Au present in POM and lysine-functionalized samples was used for comparison. All the experiments were performed in triplicates and repeated twice to obtain statistically significant results.

To quantify the amount of W or Mo present in bacterial cells after their interaction with different nanomaterials, fresh overnight grown bacterial cells (OD - 0.1 equivalents to 10^8^ CFU/mL) were independently incubated with 10 μM concentrations of different nanomaterials for 6 and 9 h at 37 °C. After incubation, bacterial cells were centrifuged at 2,000 rpm for 15 min, and the bacterial pellet was digested overnight in aqua regia and subjected to inductively coupled plasma mass spectroscopy (ICP-MS) studies for quantification of W or Mo present in bacteria.

### Cytotoxicity evaluation of surface-functionalized AuNPs in A549 human lung carcinoma cells

For the cytotoxicity assessment of nanoparticles, MTS assay was performed using a commercial kit as per the supplier’s protocol (Promega, USA). Briefly, 1 x 10^4^ A549 human lung carcinoma cells per mL were seeded in a flat bottomed 96-well tissue culture plate in the exponential growth phase and incubated for 24 h at 5% CO_2_ and 37 °C in DMEM medium supplemented with 10% fetal bovine serum and antibiotics (Life Technologies Pty Ltd, USA). A series of dilutions of AuNPs^Tyr^, AuNPs^Tyr@PTA^ and AuNPs^Tyr@PTA-Lys^ were made and added to the cells with final W concentrations of 1, 2.5, 5 and 10 µM. After 24 h of incubation, 10 µL MTS solution was added to each well and the formazan crystals thus formed were dissolved in 100 µL of detergent solution provided with the kit. The plates were read at 595 nm in a multi-scan microplate reader (Perkin Elmer, USA). Wells with complete medium, respective nanoparticles, and MTS, but without cells were used as blanks for each tested concentration. All experiments were performed three times in quadruplets, and the average of all the experiments has been shown as a cell-viability percentage in comparison with the control experiment, while nanoparticles untreated controls were considered as 100% viable. 

### Nanomaterial characterization

AuNPs^Tyr^ were thoroughly characterized at different stages of synthesis and functionalization steps using spectroscopy tools such as UV-visible, Fourier transform infrared (FTIR), X-ray photoelectron (XPS), atomic absorption (AAS) and inductively coupled plasma mass (ICP-MS) spectroscopy. Additionally, transmission electron microscopy (TEM), dynamic light scattering (DLS), and zeta potential measurements were performed on AuNPs, and nano-scanning electron microscopy (Nano-SEM) was used to observe morphological changes in bacterial cells before and after treatments. UV-vis spectral analysis was performed using Varian Cary 50 spectrophotometer operated at a resolution of 2 nm; FTIR spectra were recorded in diffuse reflectance spectroscopy (DRS) mode using Perkin-Elmer D100 spectrophotometer with a resolution of 4 cm^-1^; and XPS analysis was performed using a THERMO K-Alpha XPS instrument at a pressure better than 1 x 10^-9^ Torr (1 Torr = 1.333 × 10^2^ Pa). AAS analysis of nanoparticles was performed to find Au content using a Varian AAS spectrophotometer after dissolution of samples in aqua regia. ICP-MS analysis was carried out using Agilent Technologies 7700 series ICP-MS machine to analyze Mo or W concentration in relevant samples. Zeta potential measurements were performed both in the deionized water (pH 6.4) and the LB medium (pH 7.2) using a Malvern 2000 Zetasizer after filtering solutions through a 0.22 µm filter. TEM imaging of nanoparticles was carried out after drop casting the samples on to a carbon coated copper grid, using a JEOL 1010 TEM instrument operated at an accelerating voltage at 100 kV. Morphological changes in bacterial cells before and after treatments were visualized by FEI Nova nano-SEM. For nano-SEM imaging, samples were mounted on glass cover slip on an Al stub using double-sided carbon tape. Before imaging, all samples were coated with a 20 Å thick Pt film using precision etching coating system (Gatan model 682) to minimize sample charging during imaging. The coatings were done at 20° rock angle, 40° per sec speed, 25 rpm rotation and 5 keV beam current. The coated samples were examined using an electron acceleration voltage of 15 kV.

## Results and Discussion

**Figure 1:**
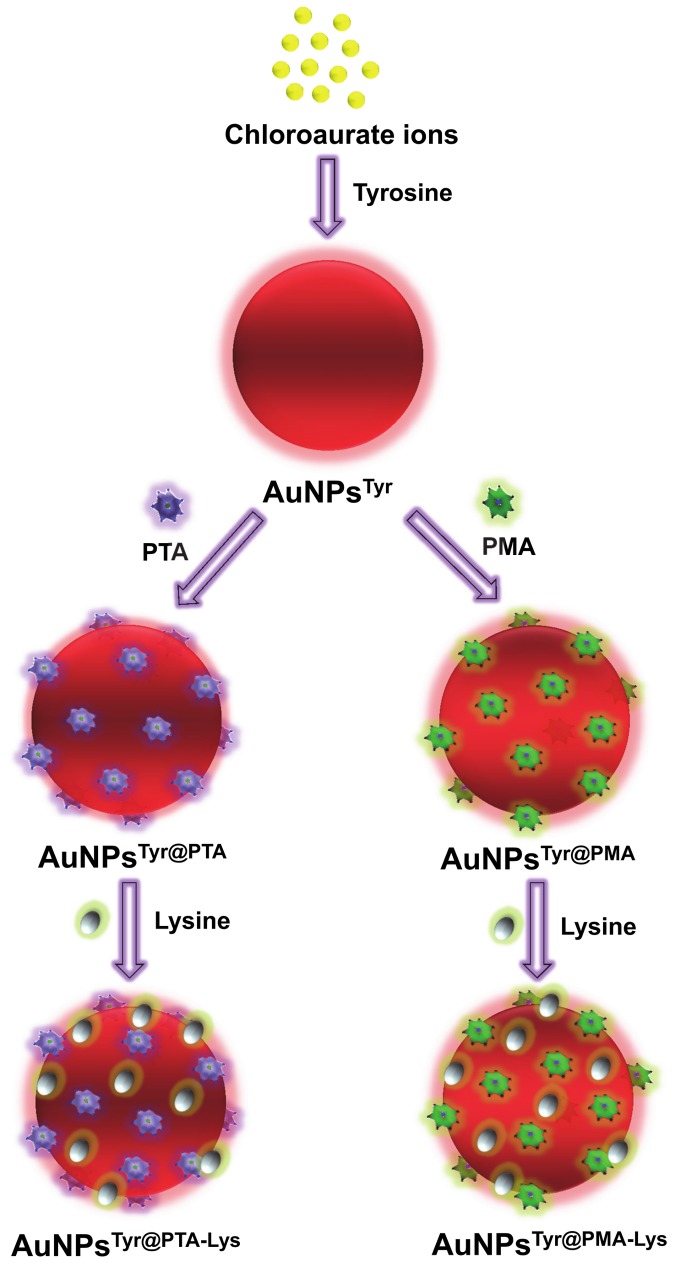
Schematic representation of tyrosine-mediated synthesis of gold nanoparticles (AuNPs^Tyr^), followed by their sequential surface functionalization with POMs (PTA or PMA) and lysine (Lys).

Figure 1 illustrates the different steps involved in the synthesis of various nanomaterials used in this study. Initially, AuCl_4_^-^ ions are reduced using tyrosine amino acid under alkaline conditions to form tyrosine-capped AuNPs^Tyr^. Under alkaline conditions, phenolic group of tyrosine acts as a reducing functional group [24], which assists in reduction of AuCl_4_^-^ ions to form AuNPs^Tyr^, and during this reduction process oxidized tyrosine molecules act as capping agent to stabilize AuNPs^Tyr^ in the aqueous solution. We noticed that the pH of the extensively dialyzed solution containing AuNPs^Tyr^ was 8.4, which is well above the isoelectric point of tyrosine (pI~5.66). Therefore, in principle, AuNPs^Tyr^ should have a negative surface charge, which was confirmed by the zeta potential value of -29 mV (Table 1). In the next step, AuNPs^Tyr^ were separately functionalized with two different POMs viz. PTA and PMA to obtain AuNPs^Tyr@PTA^ and AuNPs^Tyr@PMA^, respectively. Since POMs are highly acidic in nature, when PTA or PMA molecules were added to AuNPs^Tyr^, the pH of these solutions immediately dropped significantly to ca. 1.5-2.0, which remained 3.7 and 4.2, respectively, for AuNPs^Tyr@PTA^ and AuNPs^Tyr@PMA^ even after extensive dialysis. Since these pH values are well below the pI of tyrosine, it is expected that as soon as POMs are added to the solutions containing AuNPs^Tyr^, the surface of AuNPs^Tyr^ switches to a highly positively charged state due to the protonation of tyrosine amine (–NH_3_^+^) and carboxylic (–COOH) groups. This enables highly negatively charged POM molecules (POMs are considered as strongest heteropolyacids) to efficiently bind electrostatically to AuNPs^Tyr^, resulting in AuNPs^Tyr@PTA^ and AuNPs^Tyr@PMA^ with zeta potential values of -35.5 and -46.3, respectively. It should also be noted that since the pKa of the carboxylic groups of tyrosine molecules is 2.2, when the AuNPs^Tyr@PTA^ and AuNPs^Tyr@PMA^ attain the post-dialysis pH values of 3.7 and 4.2, respectively, some of the tyrosine molecules are expected to attain zwitterionic state in these conjugates at these pH values, wherein all the amine groups will be in protonated stage (–NH_3_^+^) and carboxylic groups will be present both in –COO^-^ and –COOH forms. This might lead to the rearrangement of tyrosine and POM molecules on the surface of AuNPs during dialysis, wherein hydrogen bonding between tyrosine and POMs may also play a contributory role in this molecular rearrangement on the AuNP surface. In the next step of sequential surface functionalization, when lysine amino acid (pI~9.74) was introduced to the solutions containing AuNPs^Tyr@PTA^ and AuNPs^Tyr@PMA^, the pH of the solutions returned back to 6.1 and 6.4, respectively (post-dialysis). These pH values are significantly below the pI of lysine; therefore amine groups of lysine will be protonated at these pH values that will enable lysine molecules to efficiently functionalize negatively charged AuNPs^Tyr@PTA^ and AuNPs^Tyr@PMA^ to form AuNPs^Tyr@PTA-Lys^ and AuNPs^Tyr@PMA-Lys^, respectively. This is evident from a reduction in negative zeta potential values from -35.5 mV to -30.9 mV in case of AuNPs^Tyr@PTA-Lys^ and from -46.3 mV to -17.9 mV in case of AuNPs^Tyr@PMA-Lys^. However, it should again be noted that the solution pH of lysine-functionalized materials is closer to the pI of tyrosine, which is likely to result in both the negatively and positively charged functional groups of tyrosine residues (zwitterionic state) to be activated at this pH. This may also potentially result in further rearrangement of the POM and lysine molecules, wherein due to the highly cationic nature of lysine, it is likely that some of the lysine molecules get sequestered between tyrosine and POMs. The possibility of molecular rearrangement in the surface-functionalized nanoparticle corona is further supported from the quantification of tungsten (W from PTA) or molybdenum (Mo from PMA) in different samples for fixed amount of Au, which indicates that 27% w/w W (PTA molecules) or 56% w/w Mo (PMA molecules) were lost during lysine functionalization of AuNPs^Tyr@PTA^ and AuNPs^Tyr@PMA^, respectively (Table 1). Therefore, although it may appear from the zeta potential measurements that these sequentially functionalized nanomaterials bear an overall negative charge at the physiological pH (at which antimicrobial tests were performed), the surfaces of these nanomaterials are rather complex and it is highly likely that the amine groups of lysine will be protonated within this complex surface environment and may rather act as guiding molecules to facilitate their uptake by negatively charged bacterial cells. Moreover, since the underlying aim of this study is to investigate the sequentially-functionalized nanomaterials for biological applications, zeta potential measurements of different nanomaterials (AuNPs^Tyr^, AuNPs^Tyr@PTA^, AuNPs^Tyr@PMA^, AuNPs^Tyr@PTA-Lys^ and AuNPs^Tyr@PMA-Lys^) were also performed in the LB bacterial growth medium (pH 7.2). This resulted in zeta potential values of -27.6 mV, -36.2 mV, -31.2 mV, -47.1 mV and -18.5 mV, respectively, which are very close to those observed in the deionized water (pH 6.4), suggesting that sequentially-functionalized materials remain stable during biological assessment. The high stability of AuNPs^Tyr^ in phosphate buffer saline (PBS) in the presence and absence of serum is also evident from the UV-visible absorbance spectra shown in the supporting information (Figure S1). 

**Table 1:** AAS (for Au), ICP-MS (for W and Mo), zeta potential, particle size and hydrodynamic radii measurements of AuNPs^Tyr^, AuNPs^Tyr@PTA^, AuNPs^Tyr@PTA-Lys^, AuNPs^Tyr@PMA^ and AuNPs^Tyr@PMA-Lys^.

Sample Name	Metal concentration (ppm)			Zeta potential (mV)	Mean particle size (diameter / SD)(nm)	Hydro-dynamic radius (nm)
	Au	W	Mo			
AuNPs^Tyr^	100.0	-	-	-29.0	6.001 / 0.85	14.6
AuNPs^Tyr@PTA^	100.0	50.2	-	-35.5	6.157 / 0.87	24.1
AuNPs^Tyr@PTA-Lys^	100.0	36.6	-	-30.9	6.145 / 0.99	18.7
AuNPs^Tyr@PMA^	100.0	-	6.8	-46.3	6.152 / 0.87	23.3
AuNPs^Tyr@PMA-Lys^	100.0	-	3.0	-17.9	6.069 / 0.93	16.7

**Figure 2:**
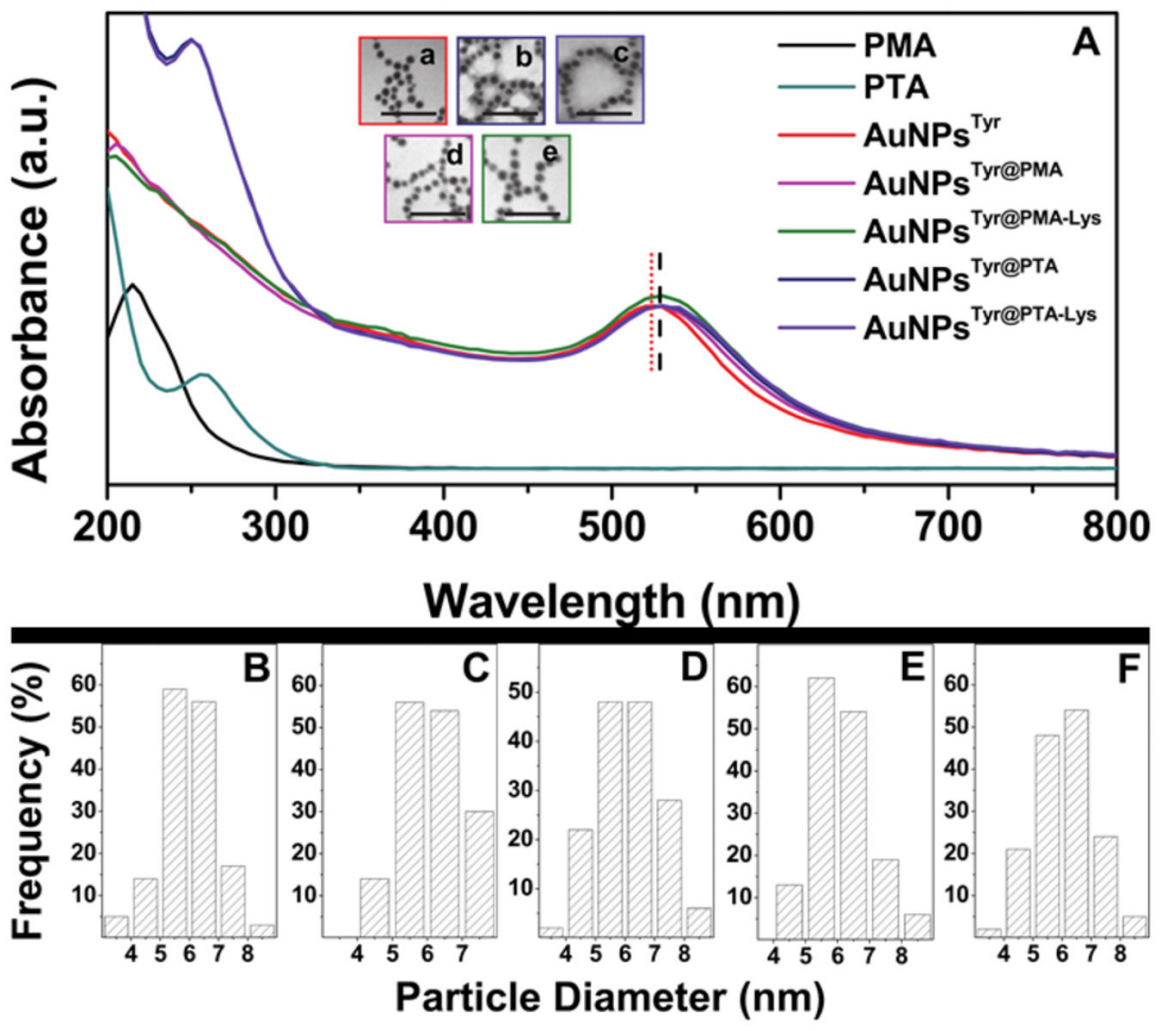
UV-visible absorbance spectra (A) and TEM images of AuNPs^Tyr^ (a), AuNPs^Tyr@PTA^ (b), AuNPs^Tyr@PTA-Lys^ (c), AuNPs^Tyr@PMA^ (d) and AuNPs^Tyr@PMA-Lys^ (e) with scale bars of 50 nm. The UV-vis absorbance spectra of pristine PTA and PMA molecules are also shown. Particle size distribution histograms (B-F) correspond to TEM images shown in (a-e), respectively.

Illustrated in Figure 2A are the UV-visible absorbance spectra of AuNPs at different stages of surface functionalization. Pristine AuNPs^Tyr^ showed a surface plasmon resonance (SPR) band with maximum at 525 nm, which shifted red to 530 nm after functionalization with POMs in AuNPs^Tyr@PTA^ and AuNPs^Tyr@PMA^, indicating binding of electron-rich POM molecules to AuNPs^Tyr^ surface. Further functionalization of these materials with lysine did not result in any spectral shifts in AuNPs^Tyr@PTA-Lys^ and AuNPs^Tyr@PMA-Lys^ and the SPR absorbance maxima for these materials remained at 530 nm. Moreover, post-POM functionalization, all the AuNPs showed blue shifts in POM absorbance maxima, for instance from 255 nm in pristine PTA to 250 nm in AuNPs^Tyr@PTA^ and AuNPs^Tyr@PTA-Lys^, and from 215 nm in pristine PMA to 205 nm in AuNPs^Tyr@PMA^ and AuNPs^Tyr@PMA-Lys^. The blue shift of POM molecules concomitant with the red shift of Au SPR further affirms the strong association of POM molecules with the AuNPs surface. TEM images corresponding to sequentially-functionalized AuNPs are depicted as insets in Figure 2A, showing that AuNPs^Tyr^ obtained using tyrosine amino acid as a reducing and capping agent are spherical in shape and highly monodispersed (less than 15% polydispersity) with an average diameter of 6 nm. Notably, AuNPs retain their monodispersity and size even after sequential surface functionalization with POMs and lysine. The particle size distribution histograms are shown in Figure 2B-F and average particle diameters obtained from TEM, as well as hydrodynamic radii obtained from DLS measurements are listed in Table 1. Since DLS provides information about the hydrodynamic radii of particles in solution, an apparent increase in AuNPs size from DLS over TEM measurements further indicates successful functionalization of AuNPs with POMs and lysine. It is also notable that although the hydrodynamic radii of AuNPs^Tyr^ increased after their functionalisation with POMs, a reduction in the hydrodynamic radii of nanoparticles was observed after lysine functionalisation step. This is most likely due to the strong electrostatic interaction between oppositely-charged POM and lysine molecules that leads to the formation of a tight organic corona around metal nanoparticles, as well as molecular rearrangement between POM and lysine molecules, thereby reducing the overall hydrodynamic radius post-lysine functionalization.

**Figure 3:**
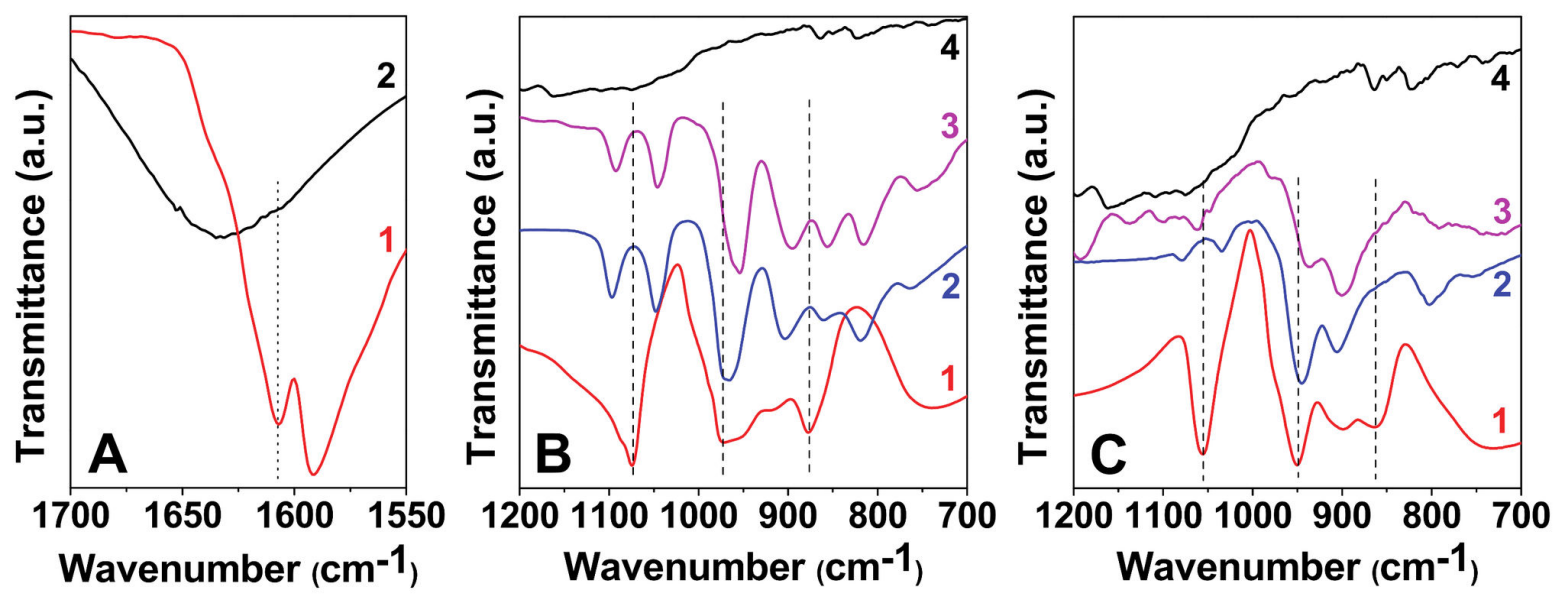
FTIR spectra in Panel A: tyrosine (1) and AuNPs^Tyr^ (2); in Panel B: PTA (1), AuNPs^Tyr@PTA^ (2), AuNPs^Tyr@PTA-Lys^ (3) and lysine (4); and in Panel C: PMA (1), AuNPs^Tyr@PMA^ (2), AuNPs^Tyr@PMA-Lys^ (3) and lysine (4).

Further, FTIR spectroscopy was employed to elucidate the functionalization of different species onto AuNPs (Figure 3). FTIR studies show that the carbonyl stretching vibration from the carboxylate ion in tyrosine shifts from 1608 cm^-1^ in case of pristine tyrosine to 1632 cm^-1^ in AuNPs^Tyr^ (Figure 3A). This shift may be attributed to the formation of a quinone type structure on the surface of AuNPs^Tyr^ due to oxidation of the phenolic group in tyrosine, while tyrosine molecules act as reducing agent for AuCl_4_^-^ ions [25]. Mode of POM binding to the AuNPs^Tyr^ was further studies by comparing the FTIR spectra arising from pristine POM molecules and AuNPs^Tyr^ with that of AuNPs^Tyr@PTA^ (Figure 3B) and AuNPs^Tyr@PMA^ (Figure 3C). The Keggin structures of POMs (PTA - H_3_PW_12_O_40_ and PMA - H_3_PMo_12_O_40_) consist of a cage of either W or Mo atoms linked by O atoms with the P atom at the center of the tetrahedra [26-29]. Oxygen atoms form distinct bonds both in case of PTA (P–O, W–O–W, and W=O) and PMA (P–O, Mo–O–Mo, and Mo=O) within their Keggin structure, which have distinguishable infrared signatures. P–O corresponds to an asymmetric stretching vibrational mode at the center of the Keggin structure; W–O–W (or Mo–O–Mo) corresponds to bending vibrational modes of O atoms that form a bridge between the two W (or Mo) atoms within the Keggin structure, and W=O (or Mo=O) corresponds to the asymmetric stretching of terminal O atoms. The different vibrational modes observed for pristine PTA, pristine PMA, AuNPs^Tyr@PTA^, AuNPs^Tyr@PMA^, AuNPs^Tyr@PTA-Lys^ and AuNPs^Tyr@PMA-Lys^ are shown in the (Table S1). The binding of PTA and PMA molecules to AuNPs^Tyr^ is clearly evident from the shifts observed in the vibrational modes of POM molecules in AuNPs^Tyr@PTA^ and AuNPs^Tyr@PMA^. Further functionalization of lysine molecules, leading to AuNPs^Tyr@PTA-Lys^ and AuNPs^Tyr@PMA-Lys^ is also clearly evident from additional shifts observed in the FTIR spectra of these samples. These vibrational shifts are in agreement to those previously proposed, indicating the complexation of tyrosine and lysine amino acids with POM molecules, leading to an amino acid-POM salt-like structure [30]. Therefore, FTIR spectroscopy provides strong evidence that the amino acids and POMs used for sequential functionalization of AuNPs provide a stable corona around nanoparticles. The presence of colloidal gold (Au^°^) in all the samples as well as the presence of W and Mo in samples containing PTA and PMA, respectively was further confirmed by XPS analysis (Table S2). The core level binding energies (BE) of C1s, N1s, O1s, Au4f, W4f and Mo4f obtained from XPS in different samples at the adventitious C1s BE of 285 eV correlate well with the literature values [24,31-34], confirming that AuNPs were coated with PTA and PMA molecules in the respective samples.

Once detailed physico-chemical characterization of sequentially surface functionalized AuNPs established confidently that our synthesis methodology resulted in well-controlled systems with different surface functionalities, we compared the antibacterial properties of these materials against Gram negative bacteria *E. coli*. Notably, metallic gold in its colloidal from is predominantly considered highly biocompatible [7], whereas PTA and PMA molecules are considered toxic to the cells due to the presence of toxic heavy metals in their structure (W and Mo, respectively) [20,35]. Therefore, we investigated the toxicity of sequentially functionalized material in a concentration-dependent manner, wherein the amount of W and Mo were kept constant in different samples. It should be noted that since AuNPs^Tyr^ did not contain any W or Mo, the amount of AuNPs^Tyr^ used as respective controls was equivalent to the highest amount of Au present (see details in Table S3). 

**Figure 4:**
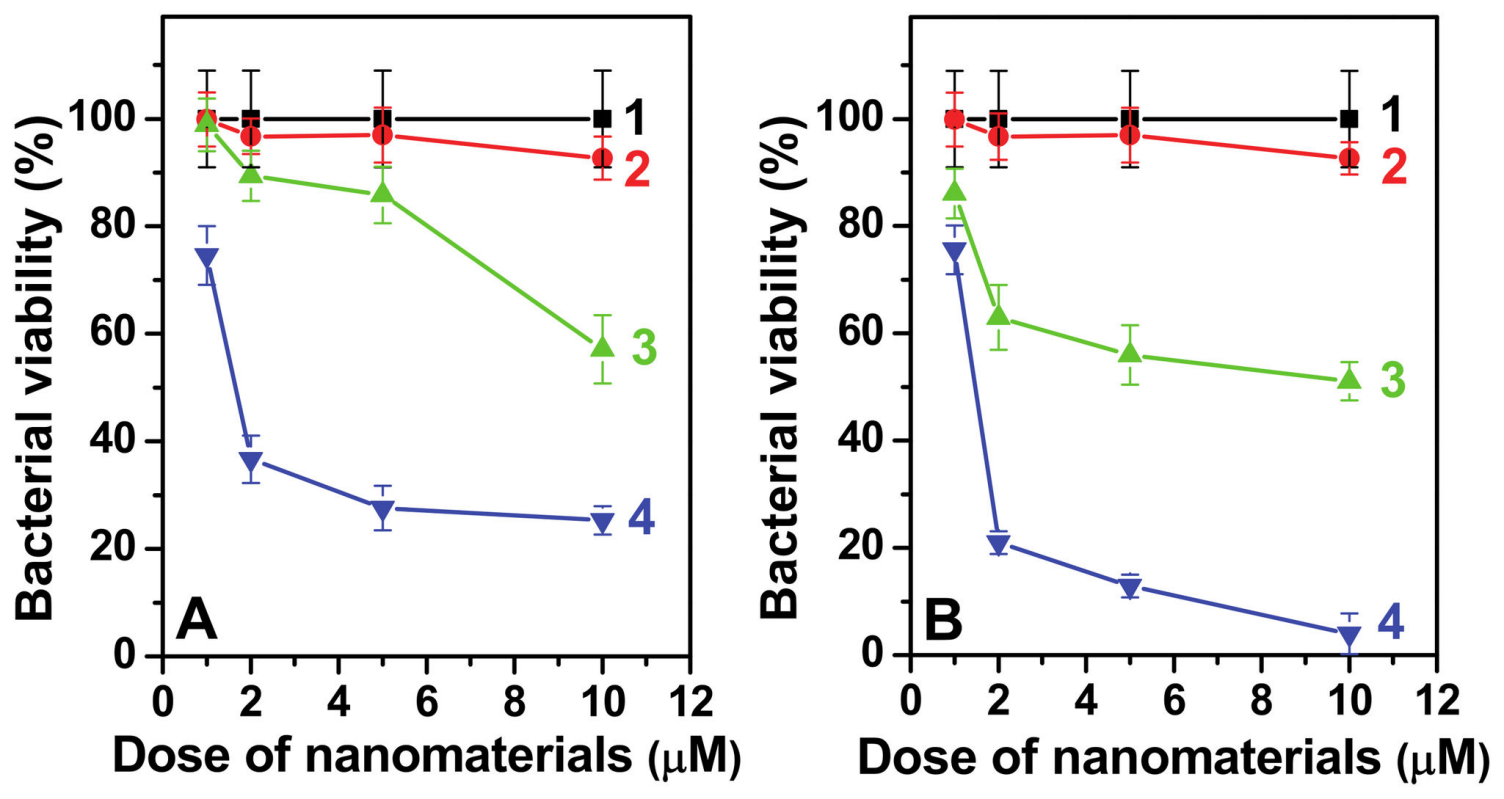
Antibacterial profile of PTA and PMA functionalized materials against *E. coli* are shown in Panels A and B, respectively. Curves 1 and 2 correspond to control bacterial cells (1) and AuNPs^Tyr^ (2), respectively. Curves 3 and 4 correspond to AuNPs^Tyr@PTA^ (3A), AuNPs^Tyr@PTA-Lys^ (a), AuNPs^Tyr@PMA^ (3B) and AuNPs^Tyr@PMA-Lys^ (4B). Doses on the x-axis correspond to either W (Panel A) or Mo (Panel B), except in curves 2, where it corresponds to equivalent amount of Au as that present in respective curves 4.

Figure 4A compares the antimicrobial performance of different AuNPs involving PTA at one of the functionalization steps while Figure 4B compares those involving PMA functionalization. AuNPs^Tyr^ were found non-toxic to the bacterial cells, indicating that surface functionalization of AuNPs with oxidized tyrosine amino acid residues during their synthesis does not significantly influence their biocompatible nature. However, surface functionalization of AuNPs^Tyr^ with POMs turned them antibacterial active and further surface modification with cationic amino acid lysine enhanced their antibacterial potential significantly. This is evident from comparing the antibacterial activity of different materials at a particular POM concentration. For instance, at a fixed W concentration of 10 µM, AuNPs^Tyr^ cause ca. 7% bacterial cell death, which increases to 43% in the case of AuNPs^Tyr@PTA^ with a further increase to more than 75% cell death by AuNPs^Tyr@PTA-Lys^. In comparison to PTA, equivalent amount of 10 µM PMA exhibited higher antibacterial potential, leading to bacterial cell death of ca. 49% and 96%, respectively when AuNPs^Tyr@PMA^ and AuNPs^Tyr@PMA-Lys^ were employed for antibacterial action. It is noteworthy that at higher tested POM concentration of 10 µM, lysine functionalization resulted in almost doubling the antibacterial potential of AuNPs^Tyr@POM^. The difference in the antibacterial profile of AuNPs^Tyr@PTA^ and AuNPs^Tyr@PTA-Lys^ at lower concentration of W (e.g. 2 µM) is even more remarkable suggesting that lysine functionalization may enhance the antibacterial efficiency by almost seven times. 

To investigate whether this increase in toxicity arises due to the cationic nature of lysine that improves the interaction of lysine-functionalized nanomaterials with the negatively charged bacterial cell wall, or whether the lysine molecules may also be inherently toxic, we performed control experiments wherein bacteria were incubated directly with lysine molecules. Pristine lysine molecules did not show significant toxicity, suggesting that the cationic nature of lysine molecules play an important role in targeting nanomaterials to the bacteria. This further supports our hypothesis that the antimicrobial profile of inherently biocompatible AuNPs can be controllably fine-tuned by their surface functionalization with appropriate molecules, as demonstrated using POMs and lysine in this study. This indicates that lysine functionalization of nanomaterials can considerably improve their antibacterial activity because due to their cationic nature, lysine can initially guide nanomaterials towards negatively charged bacterial cells through electrostatic forces, which improves the direct contact between bacterial cells and nanomaterials enabling further interaction, ultimately leading to cell death.

The direct role of lysine functionalization as a guiding molecule to achieve higher antimicrobial activity was further validated by comparing the amount of W/Mo and Au present in *E. coli*, when these cells are exposed to nanoparticles containing 10 μM equivalent of W/Mo as in the previous antimicrobial experiment shown in Figure 4 (Table S4). As discussed further, while the amount of W/Mo present in the bacterial cells could only provide the information in regards to the antimicrobial PTA/PMA present in the cells, the evaluation of the amount of Au associated with the cells provided important information in regards to bacterial uptake efficiency of different type of nanomaterials. It is clear that lysine functionalization, both in the case of PTA and PMA system, leads to an increase in the amount of POM present in the cells, which also increases as a function of exposure time (6 h vs 9 h exposure). While an increase in the exposure time from 6 to 9 h leads to a 13-21% increase in the bacterial POM concentration, the AuNPs^Tyr@POM-Lys^ samples show 16-33% enhancement in the bacterial POM concentration. Notably, Table 1 shows that the AuNPs^Tyr@PTA-Lys^ have ~10 times higher W ppm concentration than that of Mo in AuNPs^Tyr@PMA-Lys^. Hence, it is expected that in order to get the same 10 µM concentration for dosing bacterial cells for the uptake studies (Table S3), there must be a much higher AuNPs^Tyr@PMA-Lys^ density in the culture media than that of AuNPs^Tyr@PTA-Lys^. Figure 4 shows that the PMA system (Figure 4B) causes higher antimicrobial activity than the PTA system (Figure 4A). Whether this higher antimicrobial activity of PMA system is due to higher nanoparticle exposure concentration or due to higher inherent toxicity of PMA over PTA, or due to higher nanoparticle uptake of PMA system over PTA can be understood by comparing the amount of Au present in the bacteria after nanoparticle exposure. Table S4 depicts that before lysine-functionalization, both AuNPs^Tyr@PTA^ and AuNPs^Tyr@PMA^ show similar uptake by *E. coli*. This suggests that although a significantly larger proportion (~4 times – see Table S3) of AuNPs^Tyr@PMA^ are exposed to bacterial cells in comparison to AuNPs^Tyr@PTA^, their uptake is still relatively similar. It is also evident from Table S4 that lysine functionalisation of AuNPs^Tyr@PTA^ and AuNPs^Tyr@PMA^ results in different level of increase in Au uptake, wherein the AuNPs^Tyr@PMA-Lys^ is taken up in almost double in quantity over AuNPs^Tyr@PTA-Lys^. The higher degree of uptake of AuNPs^Tyr@PMA-Lys^ by *E. coli* and therefore higher cytotoxicity is most likely due to the high lysine content on these materials. The relatively high lysine content in AuNPs^Tyr@PMA-Lys^ over AuNPs^Tyr@PTA-Lys^ is further evident from zeta-potential measurements (Table 1) that show only 4.6 mV reduction in the zeta potential value of AuNPs^Tyr@PTA-Lys^ (-30.9 mV) from AuNPs^Tyr@PTA^ (-35.5 mV), whereas showing 28.4 mV reduction in the zeta potential in AuNPs^Tyr@PTA-Lys^ (-17.9 mV) from AuNPs^Tyr@PTA^ (-46.3 mV). These observations clearly confirm the role of lysine as the guiding component in achieving higher antimicrobial efficiency. 

**Figure 5:**
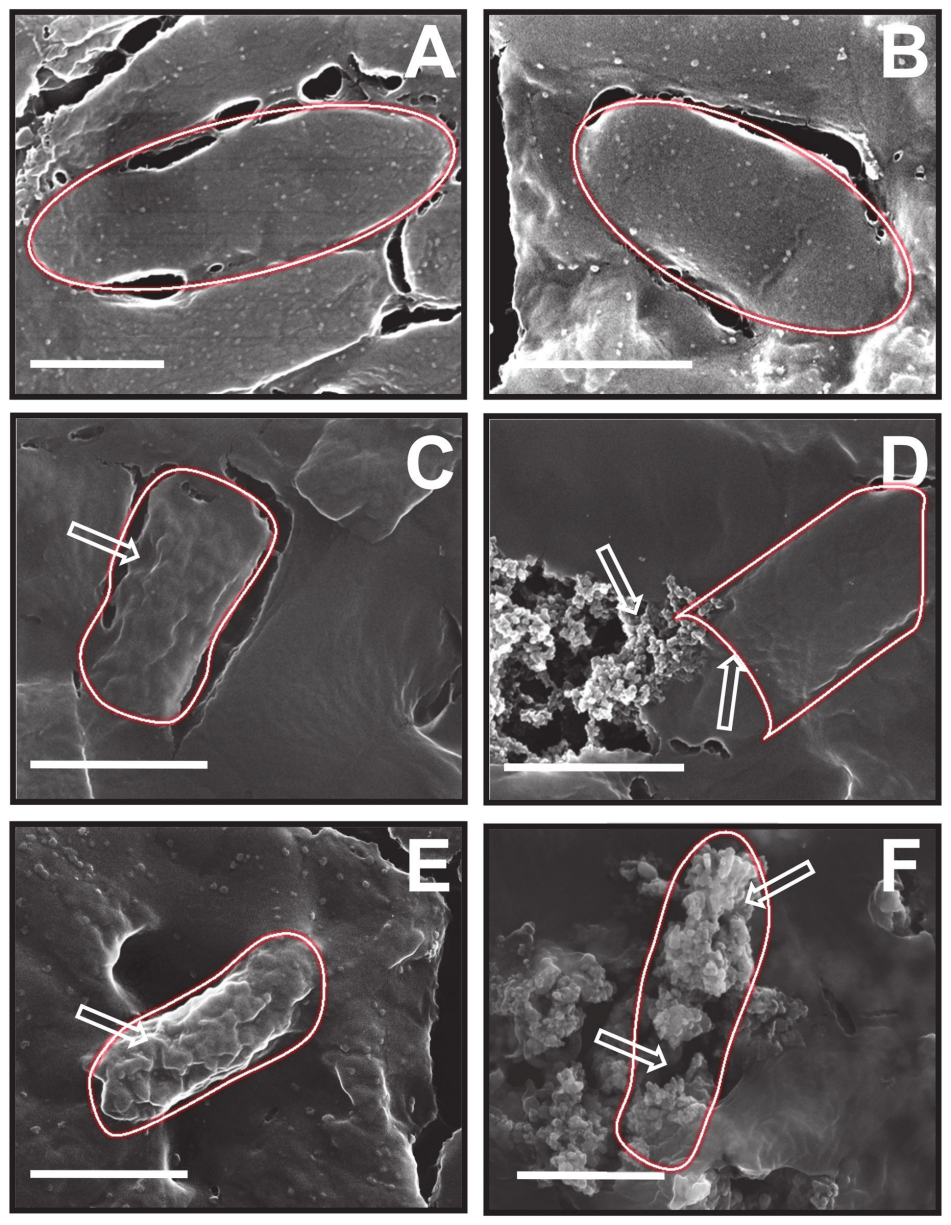
SEM micrographs of *E. coli* bacterial cells before (A) and after treatments with AuNPs^Tyr^ (B), AuNPs^Tyr@PTA^ (C), AuNPs^Tyr@PTA-Lys^ (D), AuNPs^Tyr@PMA^ (E) and AuNPs^Tyr@PMA-Lys^ (F). Scale bars correspond to 1 μm and the regions of damage in treated bacterial cells have been shown by arrows.

The mode of interaction of different materials with bacterial cell was further confirmed by monitoring the morphological changes and extent of cell wall disruption in *E. coli* using nano-SEM, wherein damaged regions in the nanoparticle-treated bacterial cells have been shown by arrows (Figure 5). The SEM images of bacteria without treatment (Figure 5A) and those treated with AuNPs^Tyr^ (Figure 5B) are alike showing an intact cell architecture with an oval morphology, thereby supporting the antimicrobial tests that AuNPs^Tyr^ are nontoxic to bacteria. However treatment of bacteria with POM- (Figure 5C and E) and lysine-modified AuNPs (Figure 5D and F) for 20 min reveal morphological changes indicative of damage to the bacterial cell integrity. The higher level of damage caused to the bacterial cells by lysine-functionalized materials in comparison to those functionalized only with POMs is also evident. For instance, AuNPs^Tyr@PTA^ (Figure 5C) or AuNPs^Tyr@PMA^ (Figure 5E) treatment of bacterial cells resulted in significant roughening of the bacterial cell (shown by arrows), suggesting the disruption of bacterial cell wall and membrane. In comparison, AuNPs^Tyr@PTA-Lys^ (Figure 5D) and AuNPs^Tyr@PMA-Lys^ (Figure 5F) treatment resulted in further damage to bacteria as indicated with arrows, wherein sub-cellular components oozing out of the bacteria due to complete disintegration of the bacterial cells are seen to such an extent that bacterial cells become indiscernible. Further, the most prominent effect of AuNPs^Tyr@PMA-Lys^ against *E. coli* in comparison to other nanoparticle systems is also evident from the comparison of these SEM images as it is observed that bacterial cells completely lose their morphological identity. From antimicrobial experiments and SEM imaging of bacterial cells, it is clear that AuNPs sequentially functionalized with POM and lysine cause irreversible bacterial cell damage and ultimate cell death by disrupting the integrity of bacterial cell wall and membrane. 

Conversely, most of the conventional antibiotics such as ciprofloxacin, doxycycline and ceftazidime act by initially penetrating into bacteria, followed by interacting with its genetic material, blocking cell-division and sometimes triggering autolysins in targeted pathogens, rather than causing physical damage to the bacterial cell wall [36]. In this mode of antibiotic action, the bacterial morphology is preserved and therefore, bacterial species may develop resistance against the antibiotic [36]. In contrast to conventional antibiotics, many cationic antimicrobial peptides do not have a specific target in bacteria and they usually interact with the bacterial cell wall through an electrostatic interaction leading to physical damage to the bacterial cells by forming pores [37]. It has been established that cationic antimicrobial peptides have the potential to overcome bacterial resistance by such a physical mode of action [38]. Since AuNPs-based antibacterial agents reported in this study appear to employ a similar physical mode of action against bacteria by causing cellular deformation leading to cell death, it is likely that such nanomaterials may offer significant opportunities to control pathogenic microorganisms by preventing them to develop resistance. It should be noted that although pristine AuNPs^Tyr^ were found to be relatively nontoxic to *E. coli*, when we previously tested the AuNPs^Tyr^ against *Staphyolococcus albus*, a Gram positive bacteria, they showed very high toxicity, leading to over 90% bacterial cell death, even without any surface functionalization [39]. Therefore, the current study involving surface functionalization using POMs and lysine could not be undertaken against Gram positive bacteria. We are currently trying to understand why AuNPs^Tyr^ showed selective toxicity against *S. albus* without showing any signs of toxicity against *E. coli*.

**Figure 6:**
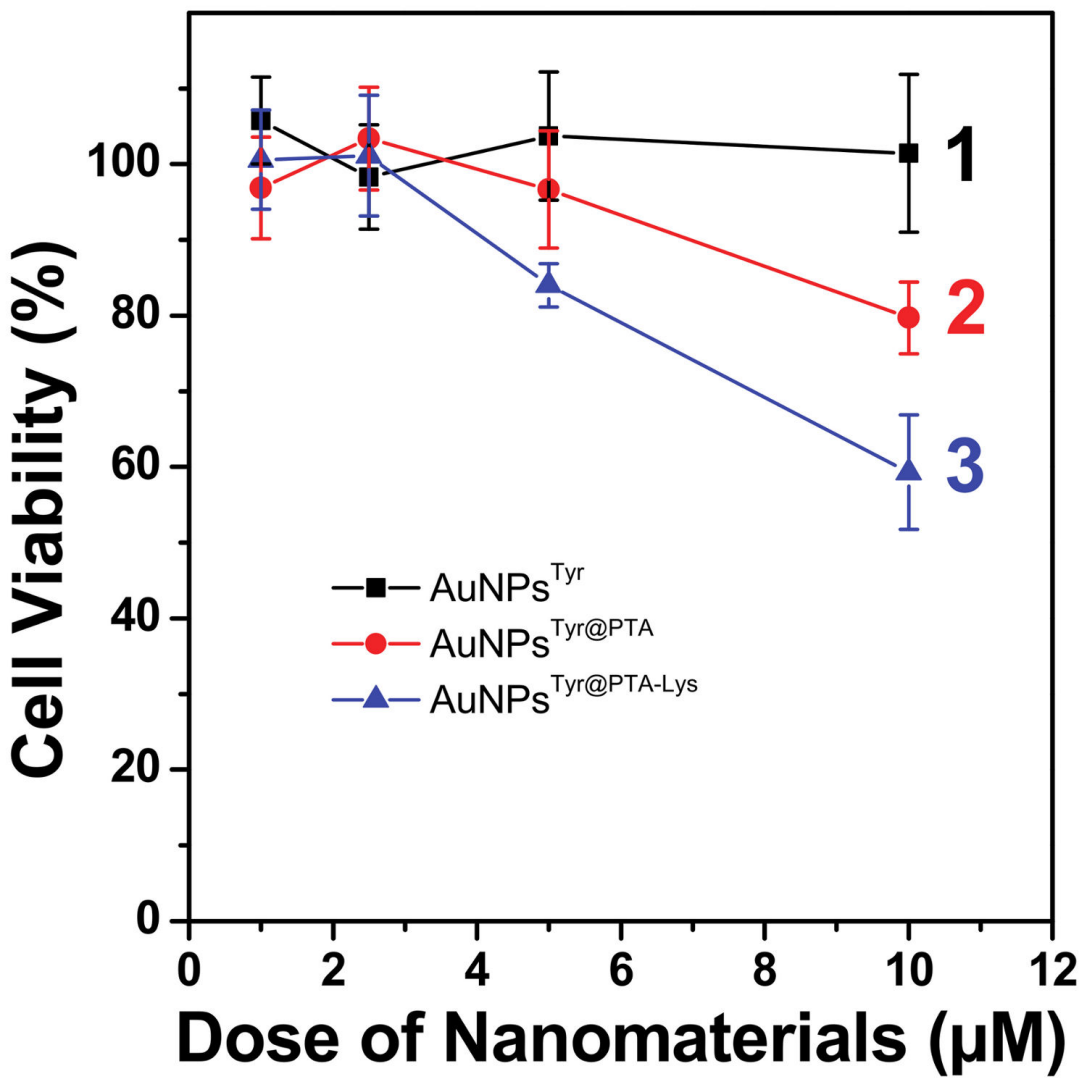
Dose-dependent cytotoxicity profile of AuNPs^Tyr^ (1), AuNPs^Tyr@PTA^ (2) and AuNPs^Tyr@PTA-Lys^ (3) against A549 human lung carcinoma cells.

Furthermore, in order to validate whether the sequential surface functionalization strategy is applicable only for antimicrobial application or it is equally applicable to mammalian cancer cells due to the presence of POM molecules in the outer corona, which are known to have anticancer activities, we also investigated the cytotoxicity of PTA-functionalized AuNPs^Tyr^ against A549 human lung carcinoma cells (Figure 6). Interestingly, although at lower concentrations (up to 2.5 µM), all the nanoparticles including AuNPs^Tyr^, AuNPs^Tyr@PTA^ and AuNPs^Tyr@PTA-Lys^ did not cause apparent cytotoxicity to the lung carcinoma cells, at higher concentrations of 5 and 10 µM, similar cytotoxicity trends as those observed during antimicrobial studies against *E. coli*, were obtained. This suggests that the negatively charged cell membranes of mammalian cells interact with these sequentially functionalized materials in a fashion similar to that with *E. coli*, wherein AuNPs^Tyr@PTA-Lys^ show the highest cytotoxicity by virtue of the ability of cationic lysine molecules to act as targeting ligands to target cancer cells, AuNPs^Tyr@PTA^ show intermediate toxicity due to anticancerous properties of POMs, and AuNPs^Tyr^ show no toxicity due to highly biocompatible nature of gold nanoparticles. However, the higher level of toxicity of these materials to *E. coli* (5 µM concentration of AuNPs^Tyr-PTA-Lys^ causes 80% bacterial death – Figure 4A) in comparison to mammalian cells (5 µM concentration of AuNPs^Tyr-PTA-Lys^ causes 20% mammalian cell death – Figure 6) indicates that these sequentially surface functionalized nanomaterials may offer potential bacterial targetability characteristics to be applied under *in vivo* settings. The applicability of these materials may include external wounds and infections, wherein a fine control between the control of bacterial infections and the growth of new mammalian cells to fill the wound is considered extremely important. The reason for higher toxicity of nanomaterials prepared in this study against *E. coli* bacterial cells in comparison to that in A549 mammalian cells is not clear at this stage. However, one of the possible factors for selective toxicity may be because *E. coli* and mammalian cells have different lysine transporter systems, which may be responsible for uptake of lysine-capped nanoparticles by *E. coli* and A549 cells with varying efficiencies, and hence difference in the level of toxicity [40-42]. However, it is not straightforward to predict whether lysine-capped nanoparticles are taken by the lysine transport systems in a way similar to that free lysine is taken up. For instance, it is now well-established that due to bulky size, nanoparticles typically enter the mammalian cells through endocytosis process, and whether this endocytosis process dominates in mammalian systems over lysine transporter-mediated active uptake, remains to be seen. Conversely, bacteria do not typically show endocytosis behavior, and therefore the role of lysine transporter systems might become more important in *E. coli* for uptake of these nanoparticles. These aspects are important to understand the underlying mode of selective antimicrobial action of the proposed materials and will be a subject of our future investigations.

## Conclusions

**Figure 7:**
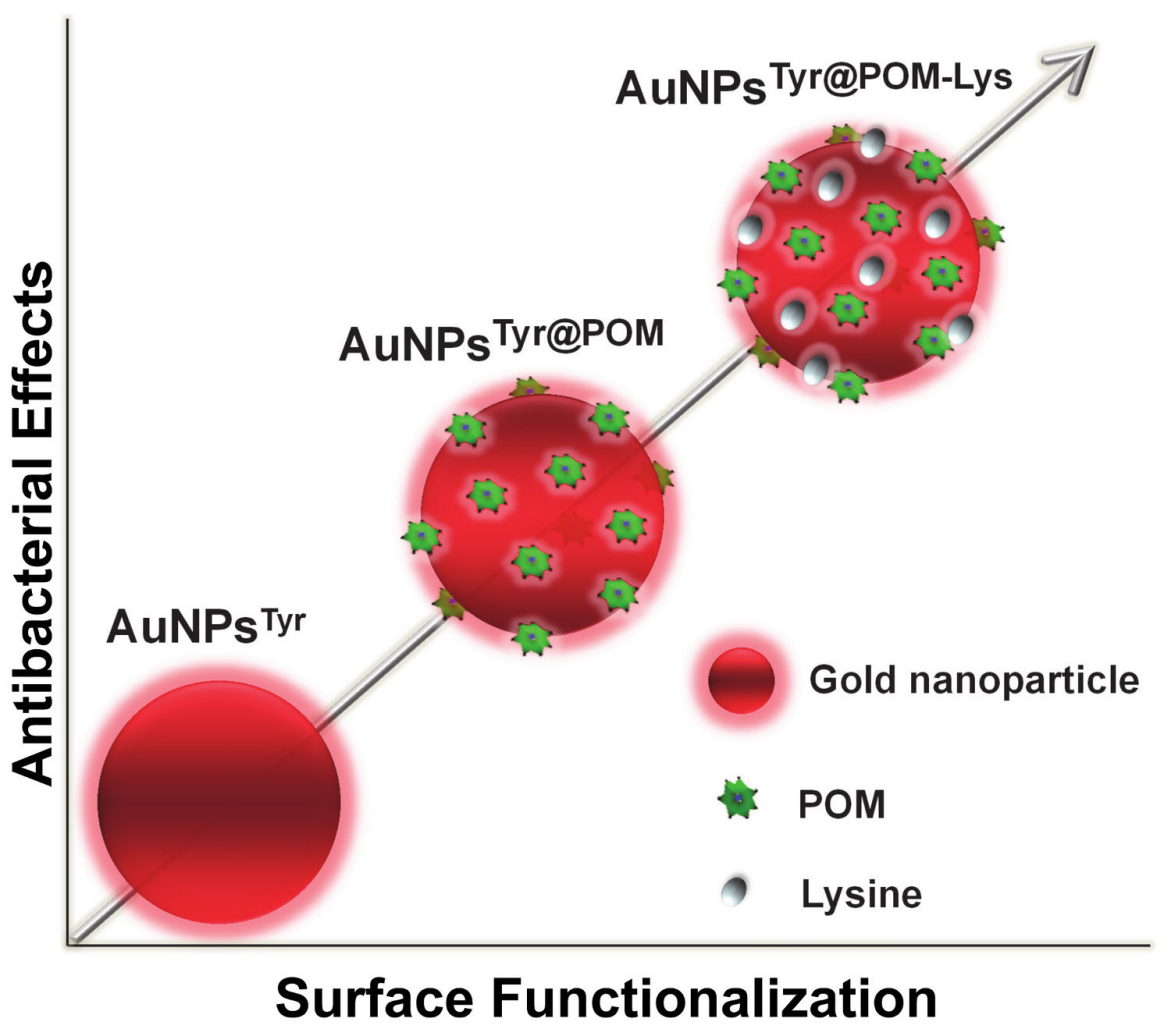
Schematic representation of the summary outcome of this work showing increase in antimicrobial activity of AuNPs after their sequential functionalization with POMs and lysine.

This study establishes the significant importance of the complex corona surrounding nanoparticles towards controlling nanomaterial properties for biological applications. In a typical nanoparticle synthesis route, different capping agents are employed during synthesis and when these materials are tested for biological applications, the observed effect is generally assigned either to nanoparticle composition, size or shape. In most of the existing studies, the effect of nanoparticle corona on biological mode of action is often neglected, if not overlooked, which is now receiving considerable attention [15]. Due to this reason, nanomaterials of similar composition, size and shapes, however those prepared using different synthesis routes show different biological profile. This study successfully employed a sequential surface functionalization approach using POMs and lysine corona onto biocompatible AuNPs to demonstrate that even highly biocompatible materials such as gold can be turned highly antimicrobial and cytotoxic by using simple ligands such as POMs and lysine through a sequential functionalization process (Figure 7). This study strongly indicates that as such, great care must be taken while assigning biological mode of action to the physico-chemical properties of different nanoparticle systems.

## Supporting Information

Figure S1 **Stability analysis of AuNPs^Tyr^ in phosphate buffer saline (PBS) in the presence and absence of serum after 24 h incubation.** No sign of aggregation of AuNPs^Tyr^ is evident from no significant shifts in the surface plasmon resonance maxima of AuNPs^Tyr^.
(PDF)

Table S1 **FTIR vibrational modes arising from PTA, PMA, AuNPs^Tyr^, AuNPs^Tyr@PTA^, AuNPs^Tyr @PTA-Lys^, AuNPs^Tyr@PMA^ and AuNPs^Tyr@PMA-Lys^.**
(PDF)

Table S2 **XPS binding energies of core levels present in AuNPs^Tyr^, AuNPs^Tyr@PTA^, AuNPs^Tyr@PTA-Lys^, AuNPs^Tyr@PMA^ and AuNPs^Tyr@PMA-Lys^.**
(PDF)

Table S3 **Concentrations of Au and W/Mo in AuNPs^Tyr^, AuNPs^Tyr@PTA^, AuNPs^Tyr@PTA-Lys^, AuNPs^Tyr@PMA^ and AuNPs^Tyr@PMA-Lys^ samples used for antimicrobial studies.**
(PDF)

Table S4 **Concentrations of Au and W/Mo in 10^8^ bacterial cells after their exposure to AuNPs^Tyr@PTA^, AuNPs^Tyr@PTA-Lys^, AuNPs^Tyr@PMA^ and AuNPs^Tyr@PMA-Lys^ for 6 and 9 h while using 10 μM equivalent of W/Mo as shown in last two columns of Table S3.**
(PDF)
