# Supplementary material for: Fine-Tuning the Antimicrobial Profile of Biocompatible Gold Nanoparticles by Sequential Surface Functionalization Using Polyoxometalates and Lysine
Source: PLoS One. 2013 Oct 17;8(10):e79676. doi: 10.1371/journal.pone.0079676 (PMC3798406; doi:10.1371/journal.pone.0079676)

Figure S1. Stability analysis of AuNPs^{Tyr} in phosphate buffer saline (PBS) in the presence and absence of serum after 24 h incubation. No sign of aggregation of AuNPs^{Tyr} is evident from no significant shifts in the surface plasmon resonance maxima of AuNPs^{Tyr}.

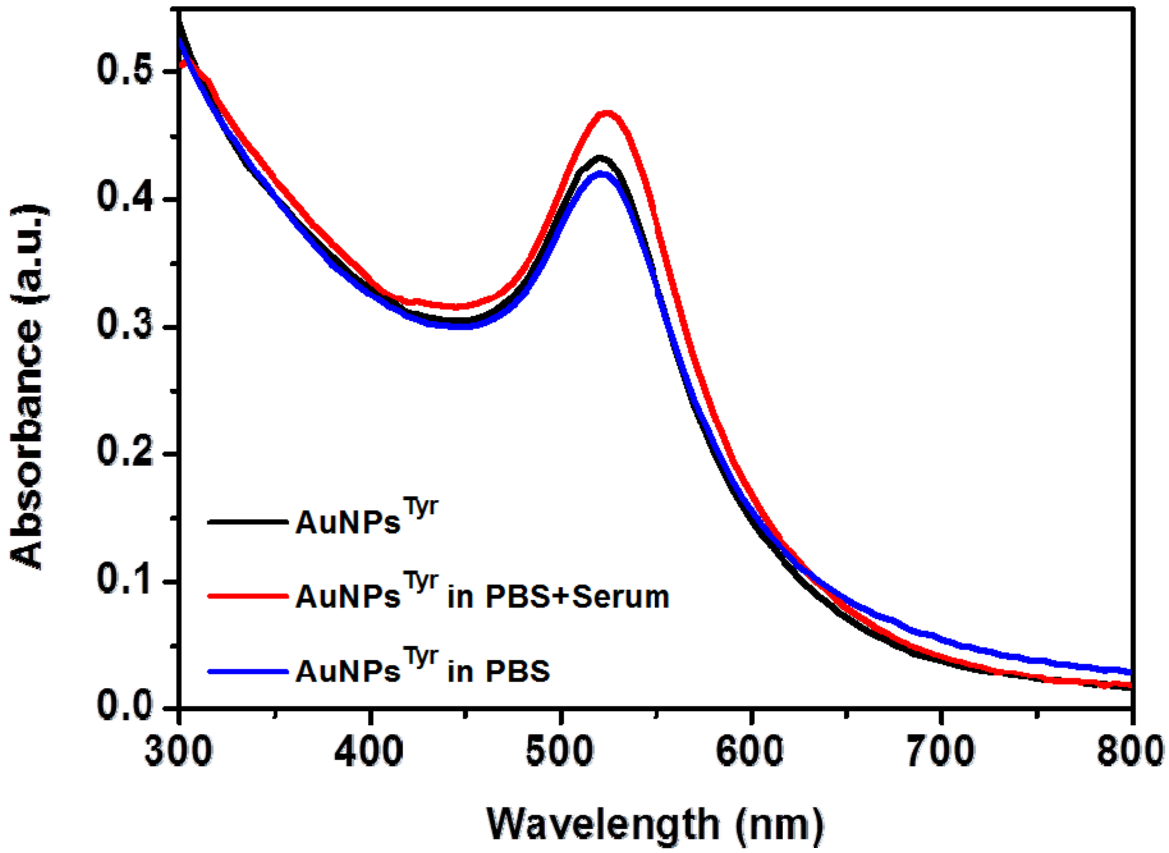

Supplement: Figure S1 — Stability analysis of AuNPsTyr in phosphate buffer saline (PBS) in the presence and absence of serum after 24 h incubation. No sign of aggregation of AuNPsTyr is evident from no significant shifts in the surface plasmon resonance maxima of AuNPsTyr. (PDF) [file pone.0079676.s001.pdf]
