# Supplementary material for: Fine-Tuning the Antimicrobial Profile of Biocompatible Gold Nanoparticles by Sequential Surface Functionalization Using Polyoxometalates and Lysine
Source: PLoS One. 2013 Oct 17;8(10):e79676. doi: 10.1371/journal.pone.0079676 (PMC3798406; doi:10.1371/journal.pone.0079676)
Supplement: Table S1 — FTIR vibrational modes arising from PTA, PMA, AuNPsTyr, AuNPsTyr@PTA, AuNPsTyr @PTA-Lys, AuNPsTyr@PMA and AuNPsTyr@PMA-Lys. (PDF) [file pone.0079676.s002.pdf]

Table S1. FTIR vibrational modes arising from PTA, PMA, $AuNPs^{Tyr}$, $AuNPs^{Tyr@PTA}$, $AuNPs^{Tyr\ @PTA\text{-}Lys}$, $AuNPs^{Tyr@PMA}$ and $AuNPs^{Tyr@PMA\text{-}Lys}$.

**Sample Name**	**FTIR Signatures (cm^{-1})**		
	**P-O**	**Mo-O**	**Mo-O-Mo**
**PMA**	1058	951	864
**$AuNPs^{Tyr@PMA}$**	1078	944	849
**$AuNPs^{Tyr@PMA\text{-}Lys}$**	1062	938	850
	**P-O**	**W-O**	**W-O-W**
**PTA**	1075	972	874
**$AuNPs^{Tyr@PTA}$**	1097	966	861
**$AuNPs^{Tyr@PTA\text{-}Lys}$**	1093	954	855
