# Supplementary material for: Fine-Tuning the Antimicrobial Profile of Biocompatible Gold Nanoparticles by Sequential Surface Functionalization Using Polyoxometalates and Lysine
Source: PLoS One. 2013 Oct 17;8(10):e79676. doi: 10.1371/journal.pone.0079676 (PMC3798406; doi:10.1371/journal.pone.0079676)
Supplement: Table S2 — XPS binding energies of core levels present in AuNPsTyr, AuNPsTyr@PTA, AuNPsTyr@PTA-Lys, AuNPsTyr@PMA and AuNPsTyr@PMA-Lys. (PDF) [file pone.0079676.s003.pdf]

Table S2. XPS binding energies of core levels present in $AuNPs^{Tyr}$, $AuNPs^{Tyr@PTA}$, $AuNPs^{Tyr@PTA\text{-}Lys}$, $AuNPs^{Tyr@PMA}$ and $AuNPs^{Tyr@PMA\text{-}Lys}$.

Sample	Binding Energy (eV)					
	**Au4f**	**W4f**	**Mo4f**	**C1s**	**N1s**	**O1s**
**$AuNPs^{Tyr}$**	83.63<br>87.53	-	-	285.00<br>287.32<br>293.33	399.65	532.18
**$AuNPs^{Tyr@PTA}$**	83.46<br>87.36	35.81<br>37.91	-	285.00<br>286.82<br>288.96	-	531.00<br>532.79
**$AuNPs^{Tyr@PTA\text{-}Lys}$**	83.66<br>87.56	35.86<br>37.96	-	285.00<br>286.58<br>288.55	399.96	531.57<br>530.76<br>533.42
**$AuNPs^{Tyr@PMA}$**	83.33<br>87.23	-	231.83<br>235.03	285.00<br>287.66<br>288.09	398.01	531.33<br>533.01
**$AuNPs^{Tyr@PMA\text{-}Lys}$**	83.65<br>87.55	-	232.46<br>235.51	285.00<br>286.55<br>288.17	399.46	531.98<br>535.42
