# Supplementary material for: Fine-Tuning the Antimicrobial Profile of Biocompatible Gold Nanoparticles by Sequential Surface Functionalization Using Polyoxometalates and Lysine
Source: PLoS One. 2013 Oct 17;8(10):e79676. doi: 10.1371/journal.pone.0079676 (PMC3798406; doi:10.1371/journal.pone.0079676)
Supplement: Table S3 — Concentrations of Au and W/Mo in AuNPsTyr, AuNPsTyr@PTA, AuNPsTyr@PTA-Lys, AuNPsTyr@PMA and AuNPsTyr@PMA-Lys samples used for antimicrobial studies. (PDF) [file pone.0079676.s004.pdf]

Table S3. Concentrations of Au and W/Mo in $AuNPs^{Tyr}$, $AuNPs^{Tyr@PTA}$, $AuNPs^{Tyr@PTA\text{-}Lys}$, $AuNPs^{Tyr@PMA}$ and $AuNPs^{Tyr@PMA\text{-}Lys}$ samples used for antimicrobial studies.

Sample Name	Metal concentrations (µM) used for antibacterial applications							
	Au	W/Mo	Au	W/Mo	Au	W/Mo	Au	W/Mo
$AuNPs^{Tyr}$	16.25	0	32.50	0	81.25	0	162.5	0
$AuNPs^{Tyr@PTA}$	1.86	1	3.72	2	9.3	5	18.6	10
$AuNPs^{Tyr@PTA\text{-}Lys}$	2.55	1	5.10	2	12.75	5	25.5	10
$AuNPs^{Tyr@PMA}$	7.24	1	14.48	2	36.2	5	72.4	10
$AuNPs^{Tyr@PMA\text{-}Lys}$	16.04	1	32.08	2	80.2	5	160.4	10
