# Supplementary material for: Fine-Tuning the Antimicrobial Profile of Biocompatible Gold Nanoparticles by Sequential Surface Functionalization Using Polyoxometalates and Lysine
Source: PLoS One. 2013 Oct 17;8(10):e79676. doi: 10.1371/journal.pone.0079676 (PMC3798406; doi:10.1371/journal.pone.0079676)
Supplement: Table S4 — Concentrations of Au and W/Mo in 108 bacterial cells after their exposure to AuNPsTyr@PTA, AuNPsTyr@PTA-Lys, AuNPsTyr@PMA and AuNPsTyr@PMA-Lys for 6 and 9 h while using 10 μM equivalent of W/Mo as shown in last two columns of Table S3. (PDF) [file pone.0079676.s005.pdf]

Table S4. Concentrations of Au and W/Mo in 10^8 bacterial cells after their exposure to $AuNPs^{Tyr@PTA}$, $AuNPs^{Tyr@PTA-Lys}$, $AuNPs^{Tyr@PMA}$ and $AuNPs^{Tyr@PMA-Lys}$ for 6 and 9 h while using 10 µM equivalent of W/Mo as shown in last two columns of Table S3.

**Sample Name**	**Au/W/Mo concentrations (µM) observed in bacterial cells**			
	**6 hour exposure**		**9 hour exposure**	
	**Au**	**W/Mo**	**Au**	**W/Mo**
**$AuNPs^{Tyr}$**	7.56	00	8.65	00
**$AuNPs^{Tyr@PTA}$**	8.92	4.80	10.17	5.46
**$AuNPs^{Tyr@PTA-Lys}$**	14.13	5.55	16.64	6.51
**$AuNPs^{Tyr@PMA}$**	8.94	1.25	10.44	1.46
**$AuNPs^{Tyr@PMA-Lys}$**	26.1	1.61	31.62	1.95
